# Immune Modulation by Chemotherapy or Immunotherapy to Enhance Cancer Vaccines

**DOI:** 10.3390/cancers3033114

**Published:** 2011-08-05

**Authors:** Genevieve M. Weir, Robert S. Liwski, Marc Mansour

**Affiliations:** 1 Suite 411, 1344 Summer St., Immunovaccine Inc., Halifax, NS, B3H 0A8, Canada; E-Mail: mmansour@imvaccine.com; 2 Room 11-L1, Sir Charles Tupper Building, Department of Microbiology & Immunology, Dalhousie University, 5850 College St, Halifax, NS, B3H 1X5, Canada; E-Mail: rliwski@dal.ca; 3 Room 206E, Dr. D. J. Mackenzie Building, Department of Pathology, Dalhousie University, 5788 University Avenue, Halifax, NS, B3H 2Y9, Canada

**Keywords:** cancer, vaccine, chemotherapy, immunotherapy, immune-modulation

## Abstract

Chemotherapy has been a mainstay in cancer treatment for many years. Despite some success, the cure rate with chemotherapy remains unsatisfactory in some types of cancers, and severe side effects from these treatments are a concern. Recently, understanding of the dynamic interplay between the tumor and immune system has led to the development of novel immunotherapies, including cancer vaccines. Cancer vaccines have many advantageous features, but their use has been hampered by poor immunogenicity. Many developments have increased their potency in pre-clinical models, but cancer vaccines continue to have a poor clinical track record. In part, this could be due to an inability to effectively overcome tumor-induced immune suppression. It had been generally assumed that immune-stimulatory cancer vaccines could not be used in combination with immunosuppressive chemotherapies, but recent evidence has challenged this dogma. Chemotherapies could be used to condition the immune system and tumor to create an environment where cancer vaccines have a better chance of success. Other types of immunotherapies could also be used to modulate the immune system. This review will discuss how immune modulation by chemotherapy or immunotherapy could be used to bolster the effects of cancer vaccines and discuss the advantages and disadvantages of these treatments.

## 1. Introduction

Literally, chemotherapy is the use of chemicals to treat cancer. The first chemotherapeutic agents were actually derived from mustard gas in the 1940's after the discovery that those exposed during war had reduced white blood cell counts [1]. Given intravenously, this treatment provided a remarkable benefit to lymphoma patients. Over the last 70 years the number of chemicals that can be used for cancer treatment has grown substantially. The most common types of chemotherapies in use today are summarized in Table 1.

In general, the mechanisms of chemotherapy result in the death of all rapidly dividing cells, tumor and healthy alike. Most tumors have a fast growth rate and are therefore targeted preferentially, but not without some damage to by-standing healthy cells. Some of the most rapidly dividing healthy cells are leukocytes and bone marrow precursors, therefore chemotherapies are generally considered to be immunosuppressive. The crudeness of chemotherapy is both a benefit and a disadvantage. One advantage is that it is difficult for tumors to resist the widespread effects of chemotherapy, but the major detriment is that chemotherapy causes damage to healthy cells. Chemotherapy is a fine balance between tumor toxicity and general toxicity, and dosages must be carefully monitored to ensure the scales are not tipped toward the latter.

Chemotherapies are not equally effective in all patients. Slow growing tumors, or tumors arrested in growth by chemotherapy, are difficult to treat because chemotherapies target rapidly dividing cells. Patients with advanced disease may first undergo debulking surgery because the drugs are not able to penetrate large tumors. Frequently, tumors develop resistance and are no longer affected by a regiment that was previously effective [3,4]. When chemotherapy is successful, there is a risk of developing secondary malignancies caused by the chemotherapy treatment itself, particularly in younger patients [5,6]. Chemotherapy has had significant success in extending patient survival, but frequently at the price of quality of life. For a long time there were no other options for cancer treatment.

## 2. Tumor-Immune System Dynamics

Historically, a healthy immune system was deemed irrelevant for treating cancer in the context of chemotherapy [2]. However, the importance of the immune system and how it interacts with the tumor has been realized. The immune system is fully capable of killing tumor cells, but it has trouble recognizing them due to tumor-induced immune suppression [7]. Tumors have developed sophisticated mechanisms of avoidance and escape. Tumor evolution proceeds on two fronts: (1) conditioning the immune system through induced immunosuppression; and (2) adaptation to immune recognition by altering expression of surface markers. Far from being independent, the tumor and immune system evolve symbiotically, and recognition of this is the defining feature of immunotherapies.

### 2.1. Tumor Influence on the Immune System

One important mechanism tumors use to escape immune detection is by engaging the immune system's natural mechanisms to avoid self-recognition. Regulatory immune cells are a diverse group found in adaptive and innate immune cell subsets that prevent autoimmunity by suppressing self-recognizing T cells. Tumors hijack this natural mechanism to escape immune detection by secreting particular cytokines into its microenvironment to promote differentiation of many types of regulatory cells [7]. Tumor-induced immune suppression is the consequence of increased proportion of regulatory cells and coinciding reduction in the activity of effector T cells targeted towards the tumor [8]. The two main types of regulatory cells now known to be associated with this process are the CD4^+^CD25^hi^FoxP3^+^ T cells (Tregs) and myeloid-derived suppressor cells (MDSCs) [9,10].

TGF-β, produced in abundance by many types of tumor cells, promotes differentiation of naïve CD4^+^ T cells into Tregs [11]. Increased Treg frequency is correlated with poor outcome and in several animal models were Tregs were selectively depleted, tumor regression was enhanced [8,12-14]. Tregs can inhibit antigen presenting cells (APCs) by inducing upregulation of inhibitory B7-H4 molecules or directly killing them through perforin and granzyme release. They engage CD80/86 on APCs with cytotoxic T lymphocyte antigen 4 (CTLA-4), leading to T cell anergy and death. Finally, they secrete immunosuppressive cytokines IL-10 and TGF-β to preserve and spread immunosuppression within the tumor microenvironment [15].

MDSC are a heterogeneous population of precursor myeloid cells that have the ability to cause immune suppression. In healthy individuals, the MDSC population is low as myeloid progenitors differentiate normally into mature myeloid cells, but under some pathological conditions maturation is arrested at various stages and the cells take on a suppressive capacity [16,17]. Tumor-derived factors, such as pro-inflammatory cytokines IL-6 and IL-1β, promote the formation of MDSCs resulting in their accumulation in the blood, lymphoid organs and tumor [18,19]. In cancer patients, the ratio of mature DCs to immature myeloid cells in the blood is inversely proportional to the stage of disease [20,21]. In humans, MDSC are identified by expression of CD33, CD11b and IL-4Rα. In mice, MDSCs universally express CD11b and GR1, for which there is no human homolog [9]. MDSCs can be divided into two groups based on nuclear morphology, the granulocytic MDSC are polymorphonuclear whereas the monocytic are mononuclear. These two subsets may have different functions in cancer [22]. MDSCs express various other surface markers including ICAM-1, CD80 and CD15, and exhibit great variability between individuals depending on the type of tumor.

MDSCs represent a significant hurdle to therapy because of their diverse immune suppression effects, both direct and indirect. They are able to directly inhibit CD8^+^ and CD4^+^ T cells in a cell-contact dependent manner through arginine and cysteine depletion, both amino acids are essential to T cell activation [23,24]. They can also inhibit T cell function though reactive oxygen species production [25]. Monocytic MDSC elevate iNOS, which may play a role in antigen-specific T cell suppression by increasing nitrosylation of MHC [25,26]. MDSC may also inhibit through antigen-independent mechanisms, it was recently shown that they reduce expression of L-selectin on naïve T cells, preventing their circulation through lymph nodes and tumors, thereby reducing the number of active T cells [27]. MDSC also indirectly cause suppression by inducing Tregs [28]. Interestingly, Treg induction may occur through CD40 expressed on MDSCs, and it was shown before this mechanism was discovered that blocking this interaction leads to reversal of CD4^+^ T cell anergy [29,30].

Suppressive subsets of many immune cell types have been found within the tumor microenvironment, including CD8^+^ T cells, NK cells and macrophages [31-34]. This diversity alludes to the intensity of suppression maintained within tumors, and the obstacles in raising an effective immune response for tumor elimination.

### 2.2. Tumor Immune Evasion

Besides inducing immune suppression, tumors have evolved other mechanisms to avoid immune detection. Firstly, tumors down-regulate expression of MHC class I and other proteins involved in antigen presentation [35-37]. Tumors can also decrease, or shed, expression of proteins that are recognized by the immune system, this concept is called immunoediting since it describes how the immune system directly impacts tumor malignancy [38,39]. Thirdly, tumors can by-pass death mechanisms by elevating expression levels of survival factors, such as anti-apoptotic proteins (survivin, BCL-X_L_), metastatic proteins (VEGF, MMPs) and proliferation factors (EGFR, c-Myc). The transcription factor STAT3 is upregulated in a number of tumors and controls expression of some of these genes [40].

Tumors contain a heterogeneous population of cancer cells that are at various states of development, allowing it to evolve quickly in response to new stresses. Tumor cells adapt to immune recognition by down regulating expression of antigens, and can also adapt to chemotherapy by increasing expression of adenosine-triphosphate binding cassette (ABC) pumps to actively secrete intracellular drugs [41]. Ironically, a successful chemotherapy regiment can also increase the chance of reoccurrence since there is potential for a few highly resistant cells to survive treatment and seed a secondary malignancy. These cells are referred to as cancer stem cells, and have been identified as a phenotypically distinct subset in some human cancers, such as AML [42].

## 3. Cancer Vaccines

The goal of cancer vaccines is to initiate an active immune response towards a tumor. There are several types of cancer vaccines in development: adenoviral, dendritic cell, tumor cell, adoptive T cell transfer and peptide [43]. Many types of cancer vaccines have been tested in clinical trials and some do elicit de novo antigen-specific immune responses, but so far few have demonstrated significant efficacy. It had long been assumed that if only cancer vaccines could elicit a strong enough immune response they could overcome tumor induced immune suppression, but after poor clinical results of so many promising vaccines it is now being realized that immunogenicity is not enough. In addition to a strong vaccine, tumor-induced immunosuppression must be actively reduced, and this may be achieved through combination with the arsenal of chemotherapy agents already in use.

## 4. Chemo-Induced Immune Modulation

It has long been understood that chemotherapies induce immunosuppression, yet it has only been of late that the specificity through which they induce suppression has been appreciated. In 2005, cyclophosphamide was the first chemotherapeutic agent that was shown to selectively deplete a regulatory immune cell population at some doses, and has inspired research into the potential immunomodulation of other chemotherapies [44]. Chemotherapies have the potential to enhance cancer vaccine-induced immune responses by lowering the defenses of the tumor [2]. There are three mechanisms through which chemotherapies may work to do this: (1) targeting the immune system to reduce tumor-induced immune suppressive cells; (2) targeting the tumor to increase immunogenicity (increase MHC or antigen expression); (3) directly stimulating effector response by activating T cells. Any one of these effects would enhance the tumor specific immune response elicited by a vaccine, and some chemotherapies may even work through multiple mechanisms.

### 4.1. Cyclophosphamide

It was first recognized in the 1980s that low doses of cyclophosphamide (CPA) specifically inhibit a population of suppressor CD4^+^ T cells and enhance immune responses against antigens [45]. It was not until 2005 that Lutsiak *et al.* showed that CPA treatment specifically affects the CD4^+^CD25^+^ T cells (Tregs) [44]. They found that mice given a low dose of CPA had a reduced Treg population with attenuated suppressor function. The Tregs were shown to undergo apoptosis, but effector CD4^+^CD25^-^ and CD8^+^ T cell populations were not compromised. The effect was transitory, maximal Treg reduction was observed 4 days after treatment but returned to normal levels by day 10. This landmark study prompted investigation into the combined use of low dose CPA with peptide vaccines. Several reports of CPA combination therapy with various cancer vaccines have demonstrated the feasibility of this treatment in murine models [46-48]. Some have demonstrated that besides reducing Treg cells, CPA therapy can also enhance CD8^+^ T cell activation and memory development through induction of type 1 interferons [49,50].

In humans, low dose CPA treatment also selectively reduces the Treg population, but reports of its augmentation of cancer vaccines have been conflicting [51-53]. In fact, investigation into the effects of CPA and other chemotherapy treatments on the immune system has emphasized the inadequacy of murine models for cancer. Human cancers are heterogeneous in nature and are characterized by a high degree of immunosuppression. In contrast, the majority of murine tumor models rely on use of implanted cell lines that are clonotypic and after years of culture *in vitro*, have lost some of their initial immunosuppressive capabilities [54]. There are some models of spontaneously arising tumors, but the advantage to using implanted cell lines is their predictability and control. Therefore, while testing cancer immunotherapies in mice does provide some indication of their efficacy, but translation into humans is difficult.

A recent report by Tongu *et al.* looked at the combination of low dose CPA plus the anthracyline doxorubicin (DR) to therapeutically treat murine CT-26 colon carcinomas [55]. The combination of CPA (i.p.) + DR (i.t.) synergistically reduced tumor growth without vaccine therapy. The effect was shown to be T-cell dependent, since no effect was seen in nude mice, and tumor specific, it could not protect from a second challenge with a different tumor. The authors speculated that CPA treatment removed Treg suppression, enhanced CD8^+^ T cell function and that in combination with DR, which is known to induce immunogenic cell death, the tumors became immunogenic. CPA and DR were combined with a GM-CSF-secreting breast tumor cell vaccine in a small clinical study [56]. Both agents were delivered intravenously and various dose combinations were tested. In twenty-two patients who received CPA + DR and vaccination, serum levels of GM-CSF remained elevated and levels of HER2 antibodies were augmented. Clinical responses were not evaluated, but these results are promising and demonstrate how two chemotherapies with slightly different mechanisms can be combined for enhanced tumor rejection. One important caveat was the effect of CPA treatment was found to be highly dependent on dose, above 200 mg/m^2^ it was immunosuppressive. This highlights the importance of dose selection when considering the immunomodulatory effects of chemotherapy.

Recently, metronomic dosing of CPA has emerged as a promising application of this drug for immune modulation. Continuous low dose CPA treatment was initially investigated for its anti-angiogenic effect since the rapidly dividing vascular intratumor endothelium are most susceptible to treatment [57,58]. It was then demonstrated that a continual low dose schedule of CPA (50–100 mg/day, p.o.) can also specifically reduce Tregs, as well as restore effector T cell and NK cell function [51]. An attractive feature of this approach is the convenience and low toxicity, which increases patient compliance.

Besides reducing Tregs, CPA treatment can also deplete B cells, augment activation and function of DCs, and skew the development of CD4^+^ T cells towards Th1 and Th17 during recovery after CPA induced lymphodepletion [59]. Interestingly, when Liu *et al.* evaluated the effects on the tumor infiltrating cell population in mice bearing tumors and treated with low dose CPA, they found a concurrent increase in the levels of myeloid derived suppressor cells (MDSCs) with the decreased levels of Tregs [60]. This work could suggest that the desirable effects of CPA treatment on the Treg population may be offset if they actually increase the level of an alternative suppressor cell, MDSCs. However, as MDSCs are loosely defined as a heterogeneous population of progenitor myeloid cells, this could merely be a reflection of enhanced lympho-proliferation following depletion.

A recent study has provided a hypothesis as to the preferential effects of low dose CPA treatment. Zhao *et al.* found that cells, such as Tregs, which have low levels of intracellular ATP have reduced capacity to detoxify internalized CPA [61]. Defining the mechanism through which CPA can selectively effect a particular population of cells will help in designing best chemotherapy-immunotherapy dosing schedules.

### 4.2. Nucleoside Analogs: Gemcitabine & 5-Fluorouracil

A study by Liu *et al.* evaluated the tumor infiltrating cell populations in mice bearing large or small tumors after low-dose CPA treatment [60]. They did confirm that CPA reduced the CD4^+^CD25^+^ population of Tregs, and also found an increased level of GR1^+^CD11b^+^ MDSCs, suggesting that in advanced tumors CPA treatment may enhance other suppressive cells. Gemcitabine (GEM) is a nucleoside analog that reportedly suppresses MDSCs specifically and has been used to reduce tumor growth in several murine models [62,63]. Like low dose CPA, GEM treatment is also transient [64]. In murine models, GEM combination with vaccine therapy significantly reduces regulatory T cells and enhances CD8^+^ T cell activation [65,66]. Knowing that MDSC can promote Treg differentiation, GEM could potentially reduce multiple suppressor cell types with a tumor both directly and indirectly.

5-fluorouracil (5-FU), another nucleoside analog, has also been reported to specifically suppress MDSCs. A comprehensive study by Vincent *et al.* evaluated several types of chemotherapies (GEM, CPA, DR, 5-FU, paclitaxel, oxaliplatin) on MDSC in EL4 thymoma tumor bearing mice [64]. They found that 5-FU specially induced apoptosis of GR^+^CD11b^+^ MDSC, both granulcytic and monocytic subsets were equally affected. 5-FU was more potent than GEM, and in combination with CPA significantly repressed tumor growth in a T-cell dependent manner.

5-FU and GEM have also been reported to increase immunological visibility of tumors by increasing expression of TAA on their surface. 5-FU or GEM were able to synergistically enhance antibody dependent cell-mediated cytotoxic (ADCC) mediated killing of colon cancer cell lines by cetuximab (a monoclonal antibody targeting epidermal growth receptor, EGFR) by increasing expression of EGFR on tumors [67]. Similar findings have been reported in other cancer models [68,69].

### 4.3. Paclitaxel

Paclitaxel (PX) therapy is common in most standard of care regimens used today because it is efficacious in many different types of cancer [70]. PX arrests cells in mitosis by preventing microtubule formation ultimately resulting in apoptosis. Recently, PX has also been shown to have stimulatory effects on the immune system, especially at lower doses than typically used for chemotherapy [71]. Conversely, standard dose PX treatment is broadly immunosuppressive and inhibits a number of cell types involved in tumor rejection: macrophages, effector T cells and NK cells [70]. The disparity between low and high dose effects has been noted with other chemotherapeutic drugs as well [72]. Interestingly, PX has been shown to be a ligand for TLR4 on murine DCs, which may be indicative of a direct effect on the immune system [73]. PX has also been shown to enhance activation of human DCs, but independently of TLR4 binding, and this effect is partially responsible for its immune-enhancing effect [74]. Investigations by the Gabrilovich group have discovered that PX treatment of cancer cells causes up-regulation of cation-independent mannose-6-phosphate receptor on the surface of tumor cells, which increases the efficiency of Granzyme B mediated cytotoxic killing (reviewed in [75]).

Low dose PX treatment has been combined with a number of vaccine types in murine models to effectively reduce tumor growth [76-78]. Used as metronomic therapy (continuous), low dose PX is a potent inhibitor of angiogenesis and specifically down-regulates expression of VEGF-receptor 2 on endothelial cells in a murine 4T1 breast cancer model [79]. In the clinic, low dose PX has not been tested in combination with cancer vaccines, but the anti-angiogenic effects of metronomic therapy have been confirmed [80-82].

### 4.4. Platinum Based Drugs: Cisplatin and Carboplatin

The platinum based drugs, cisplatin and its less toxic analog carboplatin, are often co-administered with PX in standard chemotherapy treatments. Many clinical studies have consistently shown synergism between cisplatin or carboplatin and PX treatment [83,84]. The mechanisms contributing to the synergistic effect are unknown, but addition of a third drug (e.g., GEM or epirubicin) provides no additional benefit and may in fact interfere with primary treatment [85,86]. The mechanism underlying this combinatorial effect may have to do with the unique pathways used by platinum based drugs for import and export at the cellular level, due to the presence of the heavy metal atom [41]. It is less likely that tumors can simultaneously adapt to resisting two completely different drugs.

Carboplatin on its own has little reported evidence of an immunomodulatory effect, but recently an interesting study evaluated the effect of paclitaxel/carboplatin treatment on tumors and the immune system [87]. Preliminary studies *in vitro* showed the induction of apoptosis in SKOV3 ovarian cell lines by PX/carboplatin treatment. Treated cells were also more likely to be phagocytosed by dendritic cells which acquired activated phenotype (increased MHC II, CD80/86) and were subsequently able to prime CD8^+^ T cells *in vitro*, indicating the treatment induced immunologic death of the tumors. In the same study, blood samples were collected from 13 patients with ovarian cancer receiving primary therapy with PX/carboplatin before treatment then at regular intervals afterwards. Monitoring the levels of CD4^+^ T cell, CD8^+^ T cell and NK subsets revealed that prior to treatment patients were immunocompromised as evidenced by increased Tregs and decreased Th1, Tc1 and NK cells. A single course of PX/carboplatin treatment reversed the immunosuppression, peaking around 2 weeks after treatment before returning to pre-treatment levels. Therefore, it was suggested that 2 weeks following chemotherapy treatment would be the optimal time for secondary immunotherapy treatment, however this was not studied. This systematic study of the temporal effects on the immune system show how sensitive the timing of combination therapies can be, and how they could be planned for optimal efficacy.

PX/cisplatin treatment has been tested in combination with immunotherapy in a mouse study [88]. Lewis-lung carcinoma tumor bearing mice were treated with a standard course of PX/cisplatin followed by adoptive cell therapy with cytokine-induced killer cells (CIKs). The chemotherapy preconditioning resulted in enhanced tumor rejection which was accompanied by reduced intratumoral Tregs and increased homing of the CIKs to the tumor and spleen. Therefore, even at standard doses this chemotherapy regiment has the potential to enhance immunotherapy.

## 5. Considerations for Chemotherapy-Vaccine Combinations

Chemotherapies exert various effects on the immune system that could be exploited to enhance the efficacy of cancer vaccines. However, there are several pitfalls to consider. Chemotherapeutic regiments are not universally applied, meaning that significant differences in approach are taken depending upon the type of cancer, the stage, and patient characteristics. Adding cancer vaccines into the program introduces another layer of complexity. Indeed, several studies looking at vaccine-chemotherapy combinations highlighted the fact that chemotherapies must be carefully dosed and delivered at particular times in relation to the vaccine for optimal effect [55,56]. When using chemotherapies at doses considered suboptimal for primary treatment, unforeseen effects on tumor growth may occur. For example, it is possible that low dose chemotherapy could allow tumors more time to adapt and thus become more resistant to treatment.

Although there has been significant research combining chemotherapies and vaccines in mouse models, information from human studies is sparse. Mouse models do not accurately mimic human disease, but given the success in these models more research is justified in humans. Preliminary studies, such as the one performed by Wu *et al.* [87] in ovarian cancer patients, to characterize the effects of chemotherapy alone on human patients immunity would provide valuable information for designing chemo-vaccine combination trials.

The most attractive feature of cancer vaccines is their safety, and it must be acknowledged that combining vaccines with known toxic immunosuppressants may compromise this beneficial property. Few studies have so far reported increased adverse events associated with combined treatments, but these have been mostly performed on mice. Along this line, the potential for long lasting effects of previous chemotherapy treatments should also be examined before one considers using cancer vaccines in the clinical setting. This may be especially relevant for first-in-man studies of new cancer vaccines that are typically performed in a compassionate use setting in patients with advanced cancer who have been heavily pre-treated with multiple therapies. Owing to the active role the immune system plays in tumor clearance, it is likely that the benefits of cancer vaccines will be best observed in patients with early, untreated disease.

### 5.1. Clinical Experience with Chemotherapy-Vaccine Combinations

All types of cancer vaccines stand to benefit from chemotherapy combinations, and many have already been tested in clinical studies. Due to the complexity of these combinations (scheduling and dosing of both components, as well as cancer indication and stage), rarely are two studies the same which makes comparisons difficult. Table 2 summarizes the results of some relevant studies published recently. Gemcitabine, cyclophosphamide and dacarbazine (or temozolomide, which is metabolized to dacarbazine *in vivo* [89]) in particular have been used. Most trials do not include control arms and instead rely on historical controls. Outcomes have been varied, from no effect whatsoever [52,90] to indication of increase PFS or OS (compared to historical controls) [89,91,92]. Same have noted changes to immune response profile in terms of increased diversity in epitope recognition by T cells (*i.e.*, epitope spreading) [93] or increased cellular and humoral responses [92,94]. Importantly, no studies have reported increased safety risks due to vaccine combinations with chemotherapy.

Somewhat counterintuitive are results from recent clinical studies showing that chemotherapy after vaccination may be a better treatment schedule than chemotherapy pre-treatment or concurrent treatment. Results of a clinical study published by Antonia *et al.* indicated that patients with extensive stage small cell lung cancer were actually more responsive to second-line chemotherapy treatment after vaccination with dendritic cells transduced with wild-type p53 via adenoviral vector [95]. More recently, the TG4010 viral vector encoding MUC1 and interleukin-2 was tested in a Phase II study in NSCLC patients [96]. The two arm study compared chemotherapy (cisplatin + vinorelbine) administered concurrently with vaccination or administered after vaccination. The results of the study indicated a positive outcome for both treatment arms, but number of evaluable patients was too low to conclude a preference for either schedule. For some types of cancer vaccines, this dosing schedule may be optimal because it primes the immune system before insult with chemotherapy. However, it may not be optimal for all treatment types or indications. Leffers *et al.* reported no benefits to secondary chemotherapy in ovarian cancer patients that had previously received a p53-synthetic long peptide (SLP)® vaccine, despite observing a significant benefit to NSCLC patients [97].

## 6. Strategies for Selecting Optimal Chemo-Vaccine Combinations

**Figure 1.**
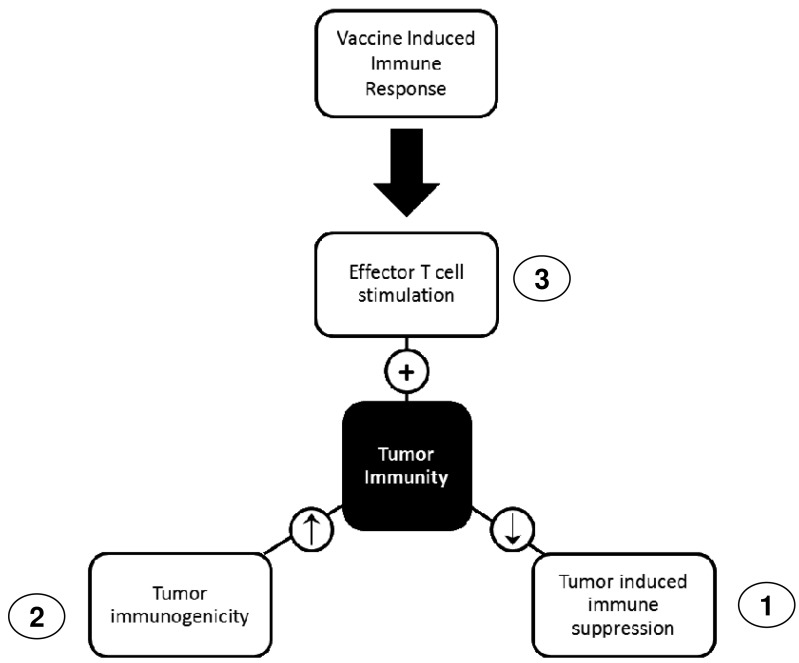
Combined effects of chemotherapy and vaccine therapy on tumor immunity. Chemotherapy can enhance cancer vaccines in three ways: (1) Reducing tumor induced immune suppression; (2) Increasing tumor immunogenicity; (3) Directly stimulating the immune system to enhance effector T cells. Chemotherapy could condition both the immune system and the tumor so that cancer vaccines have the best chance of success. Cancer vaccines focus the immune response towards the cancer and will be most effective when tumor defenses are lowered.

To overpower tumor immune evasion and suppression strategies, a successful treatment should attack the tumor from multiple angles, targeting different mechanisms quickly to minimize the chance of adaptation. To accomplish this, a targeted approach like cancer vaccines should be combined with one or more chemotherapies to help lower tumor defenses and boost the immune system. The best chemotherapies to combine with cancer vaccines would work on two levels: (1) increasing tumor visibility to the immune system through increased expression of MHC class I and unique surface antigens; (2) decreasing tumor-induced immune suppression. A third mechanism that could be exploited is the ability of some chemotherapies to increase T cell stimulation, however careful consideration must be made when combining these treatments with vaccines since this could lead to overstimulation and anergy. How these three mechanisms could work to enhance vaccine efficacy is depicted in Figure 1: vaccine-induced tumor specific T cell response could be enhanced by chemotherapies that increase T cell stimulation. Other chemotherapies can increase tumor immunogenicity, for example by increasing expression of tumor-associated antigens or MHC expression. Chemotherapies can also condition the immune system to reduce tumor-induced immune suppression, thereby allowing the vaccine-induced immune response to prevail. Examples of chemotherapies that can mediate each mechanism are given in Table 3.

CPA and PX were shown to increase effector T cell stimulation via shifting the immune response towards Th1 after vaccination with a GM-CSF-secreting whole-cell vaccine [78]. Tumor immunogenicity can be increased in several ways, DR is an example of a chemotherapy that can induce the immunologic death of tumor cells [102]. Another way to increase tumor immunogenicity is by causing upregulation of tumor-specific markers, for example 5-FU and cisplatin were shown to cause increase in tumor-antigen expression in cancer lines *in vitro*, leading to increased recognition and killing by antigen-specific CD8 T cell lines [103]. CPA and GEM are prominent types of chemotherapies that have a direct effect on discrete components of the immune system, Tregs and MDSCs respectively, and were discussed in detail in preceding sections.

Some chemotherapies can work through multiple mechanisms, for example CPA can not only reduce Tregs [44], but also increase effector T cell function [78]. Combining multiple chemotherapies is another approach to targeting different anti-tumor mechanisms, for example one study described above has demonstrated that CPA + DR is a viable combination that could potentially synergize with vaccination [55]. However, some chemotherapy combinations may not work well together, for example mitomycin C does not synergize with DR although CPA can [55]. More research should also be conducted to discover the mechanisms through which these chemicals work, and how they are selective for these pathways. For example, why does GEM only target MDSC? It is possible that GEM is in fact a growth promoter that can facilitate MDSC differentiation into a mature myeloid cell. In which case, GEM would be an optimal candidate for combination with vaccine therapy as the vaccine could guide the activation of the newly differentiated myeloid cells.

In addition to their immune modulating effects when used concurrently with immunotherapy, chemotherapies can also be utilized to increase the sensitivity of tumors to subsequent immunotherapy treatments. In this scenario, chemotherapy is used to destroy the most susceptible tumor cells and reduce tumor burden, potentially leaving behind residual cancer cells not susceptible to treatment, *i.e.* the cancer stem cells. At this point, with low tumor burden and fairly uniform cancer cell population, the patient could be treated with a cancer vaccine targeting specific proteins essential to the stem cell survival.

Another consideration in chemo/vaccine combinations could be the molecular target of the vaccine. For example, survivin is an anti-apoptotic protein that is upregulated by many types of cancers to such an extent that it has been proposed as a “universal” cancer target [105]. Several pre-clinical and clinical studies have evaluated survivin-based peptide vaccines and demonstrated variable efficacy. In addition to its role in preventing cell death, survivin is also an essential regulator of the cell cycle that binds to and stabilizes the mitotic spindle [106]. As described above, the mechanism through which PX induces tumor apoptosis is through arresting cells undergoing mitosis. Therefore, PX treatment could be complementary to a survivin-targeted vaccine since not only does it induce immunologic death of tumors, but by freezing cells in this state it could increase the expression of the vaccine target.

## 7. Antibody-Induced Immune Modulation

Chemotherapy has been the mainstay of cancer treatment for many years, but the latest breakthrough in the field is the development of monoclonal antibodies (mAb). Treatments with mAb were initially designed to target tumor cells directly and subsequently induce tumor destruction through several different mechanisms. There are in fact nine mAb of this type that have been approved for various cancer indications since 1997 [107]. Avastin, developed by Genentech/Roche, has a slightly different mechanism in that it blocks to process of angiogenesis by binding to vascular endothelial growth factor A (VEGF-A), a chemical signal over-produced by tumor cells. Some of the approved mAb are conjugated to a toxic molecule, either a chemical agent or radioactive particle, that will selectively kill the tumor cells recognized by the antibody.

Basic mechanisms through which monoclonal antibodies work include blockade of growth receptors or activation receptors, antibody-dependent cell-mediated cytotoxicity and complement mediated cytotoxicity [107]. Antibodies can also enhance tumor cell phagocytosis and tumor antigen processing by linking to Fc receptors on antigen presenting cells (APCs), thereby serving a link to induction of cellular immunity. A study by Rafiq *et al.* first demonstrated that administration of tumor-targeted antibodies not only induces T cell immunity towards the targeted epitope but also others through epitope spreading [108].

Unlike chemotherapies that have dose-dependent toxicity and are crudely tumor-selective, mAb have a relatively good safety profile and defined targets. Although effective, the main limitation of mAb therapy is applicability; they can only be used to treat cancers that express the target, and even then are generally only effective in about 30% of patients [109]. For example, trastuzumab is only applicable for breast cancer patients positive for Her2/*neu* expression, about 15–20%. Furthermore, tumors can develop resistance through the shedding of the mAb target (immunoediting).

Monoclonal antibodies can also be used for immune modulation. This type of mAb actually targets components of the immune system to enhance or block effect. For example, antibodies targeting the suppressive co-stimulatory receptors CTLA-4 or PD-1 on T cells block inhibitory signals typically transmitted through these receptors and prolong the life of activated T cells. Several mAb that target the immune system are in various stages of clinical development, summarized in Table 4. Importantly, mAb that target immune system are less likely to be rendered unusable since the immune system cannot shed the targets as tumors can. The mechanisms of mAb immunotherapy are, in theory, easier to predict than chemotherapy since the target is known, yet in practice has proven difficult due to the redundancy of the immune system and our lack of complete understanding.

### 7.1. Anti-CTLA-4 Therapy

The most developed mAb of this type target the T cell surface protein CTLA-4. CTLA-4 is a negative regulator of effector T cell activity and is induced upon activation. CTLA-4 out-competes the co-stimulation molecule CD28 for binding B7 molecules on antigen presenting cells and instead delivers an inhibitory signal [111]. Therefore, CTLA-4 is used as a braking mechanism to control T cell responses. It is also used by Tregs for immune suppression; Tregs constitutively express CTLA-4 and induce suppression to DCs when binding through B7 [112]. The DCs in turn induce apoptosis and anergy in T cells [15]. Two fully human antibodies have been developed that target CTLA-4: tremelimumab (by Pfizer) and ipilimumab (by Bristol-Myers Squibb). Potentially, these antibodies could work on two fronts, first by blocking effector T cell CTLA-4 and thereby extending their survival, and second by blocking Treg CTLA-4 to prevent this mechanism of suppression. However, studies have demonstrated that in humans anti-CTLA-4 treatment targets effector T cells only [113,114]. Ipilimumab was recently approved by the FDA for second line treatment of advanced melanoma, but both have been tested in a number of clinical trials targeting various indications, such as melanoma, and have provided positive benefit [115]. Despite being able to induce tumor regression in 10% of patients, Pfizer halted the development of tremelimumab based on a dismal increase of overall survival of only 1 year in a recent phase III trial [116].

The results of a phase III clinical trial of ipilimumab, which supported FDA approval for this mAb, were presented at the American Society of Clinical Oncology (ASCO) meeting in 2010 [117]. The 1:1:3 randomized study containing 750 patients compared ipilimumab treatment alone to vaccination with GVAX (peptide vaccine targeting the melanoma TAA gp100) and to combination treatment with both ipilimumab and GVAX. Patients who received ipilimumab alone or in combination with GVAX were not significantly different and experienced a 10% increase in 2-year survival rates and increased overall survival compared to patients who received GVAX alone. Although these results were used to approve ipilimumab treatment in advanced melanoma patients, they are somewhat controversial because GVAX alone was used as the control arm, and not the common dacarbazine treatment used for advanced melanoma patients [118]. From a vaccine perspective the results are discouraging. Other peptide vaccines targeting gp100 have shown immunogenicity in other small clinical trials, demonstrating that it is possible to break tolerance towards this TAA, yet in this study no effect was attributed to GVAX treatment [119,120]. Pre-clinical research had also indicated that murine anti-CTLA-4 could in fact synergize with peptide cancer vaccines in mice [121-123]. The advanced stage of the patients in the ipilimumab study may have been detrimental to vaccine efficacy, and could show that although ipilimumab does provide some benefit to these patients, it cannot synergize with peptide vaccines in this cohort. Notably, the authors did not report if gp100-specific T cells were raised in any group so it is unclear if the patients immune systems responded at all to vaccination [124]. It is also possible that ipilimumab cannot synergize with cancer vaccines due to the isotype of this antibody. Ipilimumab, like the majority of mAb developed to date, is IgG1 isotype, which induces moderate complement activation and strongly induces phagocytosis by binding to Fc receptors. Although this isotype is ideal for mAb targeting tumor cells for destruction, ipilimumab targeting activated T cells may inadvertently enhance their elimination. In contrast, tremelimumab is IgG2 isotype, which is a poor activator of complement and weak binder of Fc making it an ideal subclass for blocking interactions. It would be interesting to compare both anti-CLTA4 mAb in combination with vaccination to see if tremelimumab can induce a greater synergistic effect than was observed with ipilimumab. Indeed, a better understanding of which antibody isotypes synergize best with vaccines is needed for rational design of future clinical trial protocols involving these two emerging immunotherapies for cancer.

### 7.2. Anti-PD-1 Therapy

PD-1 (programmed death 1) is a member of the CD28 superfamily, like CTLA-4, and is upregulated on T cells upon activation [112]. PD-1 is a suppressive regulator of T cell activity, ligation with its receptor results in inactivation and apoptosis. The receptors for PD-1, PD-1L and PD-2L, are normally expressed on self-cells to prevent autoimmunity, however PD-1L is upregulated by a number of tumors to quell anti-tumor T cell responses [125-127]. Accordingly, tumor infiltrating CD8^+^ and CD4^+^ T cells have been shown to have increased expression of PD-1 and are anergic [128,129]. Combined treatment of anti-PD-1 treatment and a GM-CSF secreting whole cell vaccine significantly prolonged mice challenged with B16 melanoma or with CT26 colon cancer, whereas monotherapy with either treatment had no effect [130]. The combined treatment was associated with increased antigen-specific CD8^+^ T cell infiltration of the tumor. Another study by Mongsbo *et al.* also found that monotherapy with anti-PD-1 is not as effective as anti-CTLA-4 monotherapy, but together may have an additive effect in prevention of MB49 murine bladder cancer [131]. The combined blockade of both PD-1 and CTLA-4 was found to synergize with a vaccine in treating of B16-B6 melanoma tumors [132]. The synergistic effect on tumor growth was mirrored with increased tumor infiltration of CD8^+^T cells expressing CTLA-4 and PD-1, presumably without treatment these cells would have been anergized. Dual blockade of PD-1 and CTLA-4 signaling eliminates two T cell suppressive mechanisms, therefore this is a logical combination that should increase longevity of T cells. A human PD-1 antibody (MDX-1106) was recently tested in a clinical trial in patients with several types of advanced cancer [133]. In the small phase I study, 39 patients were treated with antibody monotherapy and levels of PD-1 on circulating PBMCs as well as levels of PD-L1 on tumor cells were monitored. They found that tumor expression of PD-L1 may be indicative of responsiveness to MDX-1106 treatment, but overall clinical responses were low.

An alternate, or perhaps additional, mechanism for the synergistic effect of combined PD-1 and CTLA-4 blockade is by inhibition of MDSC suppression. One group reported that MDSCs isolated from mice bearing I8D ovarian tumors had elevated levels of both PD-1 and CTLA-4 [134]. When blocking antibodies were administered *in vitro*, the MDSCs had reduced arginase I activity; arginase I is a mechanism through which MDSCs attenuate T cell activation. *In vivo* treatment of tumor bearing mice reduced tumor burden and increased survival.

### 7.3. Anti-GITR Therapy

Complementary to T cell boosting strategies with anti-CTLA-4 or anti-PD-1 would be Treg inhibition using Treg-specific antibodies. Initially, antibodies towards the relatively non-specific CD25 surface marker found on Tregs were used in an attempt to target this T cell subset. However, anti-CD25 mAb clinical trials (daclizumab by Hoffman-LaRoche) have experienced mixed results; although this antibody does deplete Tregs, it also has an effect on activated effector T cells, which also upregulate CD25 [135]. The result is too devastating on the developing anti-tumor immune response unless timed correctly, which could present technical limitations for heterogenous human patients [135,136]. A new target is GITR (glucocorticoid induced TNF receptor), a co-receptor expressed in constitutively high amounts by Tregs and also increased on activated T effectors. Interestingly, while co-stimulation of CD3 and GITR results in proliferation of both Tregs and effector T cells, the expanded Tregs become functionally unresponsive while the effector T cells gain functional activity [137]. A single administration of the murine anti-GITR antibody DTA-1 eradicates or reduces tumor growth in different mouse models [138-140]. Mice challenged with B16 tumors and treated with DTA-1 developed strong antigen-specific T cell responses, and when combined with a melanoma vaccine, DTA-1 treatment enhanced primary and recall CD8^+^ T cell responses [141,142]. The mechanisms underlying DTA-1 treatment are truly two-fold, they can both enhance effector T cells and reduce Tregs. The mechanism through which they reduce Treg function is not clear, in one study Tregs isolated from tumors of DTA-1 treated mice did not have impaired suppressive function and yet relative numbers of Tregs were reduced compared to CD4 or CD8 T cells, suggesting depletion [138]. However, some studies have found no change in the absolute number of CD4^+^ T cells after DTA-1 treatment, and no death observed *in vitro*. Instead, it has been proposed that DTA-1 treatment reduces the lineage stability of Tregs through loss of FoxP3 expression [139]. This could mean that Tregs are converted to Th17 cells, these cells are known to be reciprocally regulated and instances of Treg conversion into Th17 have been documented [143]. It would be interesting to see if this was the case with DTA-1. In any case, the combined blockade of CTLA-4 and GITR with mAb was recently shown to synergistically reduce tumor formulation in two different murine tumor models, demonstrating that their respective effects on Tregs and effector T cells, in the end, work together [144].

## 8. Considerations for Antibody-Vaccine Combinations

Antibody therapies for immune modulation are an exciting new area of discovery in immunotherapy research. As an alternative to chemotherapy immune modulation they offer a defined mechanism of action since the target is known. However, due to the redundancy of the immune system and the fact that we still do not fully comprehend its complexity, antibody therapies still carry the risk of off-target side effects. Further, since immuno-modulatory doses of chemotherapies are often low and non-toxic, antibody therapies may loose their safety-edge since they still must be used at standard doses. Obtaining relevant pre-clinical data for mAb therapy is also difficult since the human antibodies cannot be tested in common strains of mice, so we must rely on translation in models that use murine homologs of the antibodies. Several clinical trials are currently evaluating these antibody therapies in conjugation with vaccine therapy. As the results of these trials emerge, and our understanding the of the immune system increases, antibody therapies may emerge to become the standard complementary treatment to vaccines in the future of immunotherapy.

## 9. Closing Remarks

Since the proposal of a “magic bullet” for cancer treatment, researchers have been looking for the one cure that will stop all cancers. With each new development (surgery, radiotherapy and then chemotherapy) it has become increasingly obvious that the best course of treatment utilizes multiple methods. Immunotherapy is the next step in cancer care, and may also have best results when used in combination with other therapies. Different immunotherapy approaches have different strengths, vaccines elicit and guide an immune response and antibodies or chemotherapies can reverse tumor-induced immune suppression. The future of cancer therapy lies in combining these treatments effectively, which hinges on our understanding of the role of the immune system in tumor rejection. It is for this reason that cancer immunotherapy is evolving alongside our understanding of the immune system.

Whatever the approach, it is increasingly becoming apparent that the most promising cancer therapies cannot work alone. Cancer vaccines, chemotherapies and immunotherapies must be combined effectively to attack the tumor from multiple sides to quickly and thoroughly eliminate cancer.

## Figures and Tables

**Table 1.** Common chemotherapy agents and their classification (adapted from [2]).

**Type**	**Mechanism**	**Examples**
Alkylating Agents	Modification of nucleic acid functional groups	Cyclophosphamide, dacarbazine
Antimetabolites	Nucleoside analogs, perturb RNA and DNA synthesis	5-fluorouracil, gemcitabine
Taxanes	Disruption of microtubule formation, stop cell division	Paclitaxel, docetaxel
Anthracylines	Interfere with DNA replication machinery, inhibit RNA and DNA synthesis	Doxorubicin
Platinum based	Cross link DNA	Cisplatin, carboplatin, oxaliplatin

**Table 2.** Clinical reports of peptide-vaccination in combination with chemotherapy.

**Vaccine**	**Chemotherapy**	**Indication**	**Outcome**	**Ref.**
Personalized peptide vaccine (once/ week for 8 weeks)	Gemcitabine (1000 mg/m^2^, i.v.; once/ week for 3 weeks, one week off, then repeat)	Advanced pancreatic cancer	Phase II study, single arm. Response rate of 67%, both cellular and humoral responses detected	[94]
WT-1 peptide vaccine (day 8, 22)	Gemcitabine (100 mg/m^2^ on day 1, 8, 15)	Pancreatic and biliary tract	Phase I study, single arm study. Combination safe. GEM treatment increases numbers of monocytes and DCs.	[98]
Melan-A + gp100 peptide vaccine + IFN-a (day 1, 8, then every 21 days for 5 courses)	Dacarbazine (800 mg/mq i.v.; one day before each vaccination)	Melanoma	Phase I study, single arm. Dacarbazine treatment resulted in increased diversification of TCR repertoire	[93]
GV1001 (3 injections during week 2, 2 injections during week 3, single injection on weeks 6, 7 and 11)	Temozolomide (200 mg/m^2^, p.o.; 5 consecutive days every 28 days)	Advanced melanoma	Phase I study, single arm. Safe. Increased OS compared to predicted survival. Development of polyfunctional cytokine profile. Durable GV1001-specific T cell responses.	[89]
EGFRvIII vaccine (day 21 of each 28 day cycle)	Temozolomide (a) 200 mg/m^2^ for first 5 days in each cycle; (b) 100 mg/m^2^ for first 21 days in each cycle)	Newly diagnosed glioblastoma	Phase II study, 2 arm, historical controls. Compared two different dose schedules of chemotherapy. Both groups resulted in better OS than historical control. Interestingly, longer treatment (b) caused more profound and persistent lymphopenia with an increase in Tregs, yet still mounted potent cellular and humoral immunity.	[92]
GV1001 (days 1, 3, 5, 8, 15, 22, 36 followed by 4 weekly injections)	Cyclophosphamide (300 mg/m^2^ i.v.; single pre-treatment 3 days before vaccination)	Advanced HCC	Phase II study, single arm. No significant effects on immune response or tumor growth observed.	[52]
MELITAC – containing 12 melanoma CTL epitopes (days 1, 8, 15, 29, 36, 43 then month 3, 6, 9, 12)	Cyclophosphamide (300 mg/m^2^ i.v.; single pre-treatment)	Resected stage IIB to IV melanoma	Phase I/II study, 4 arms testing two vaccines with or without CPA. “Cyclophosphamide provided no detectable improvement in CD4 or CD8 T-cell responses or in clinical outcome.”	[90]
BLP25 – MUC1 peptide delivered in liposome formulation (weekly vaccinations for 6 weeks)	Cyclophosphamide (300 mg/m^2^; single pre-treated 3 days before vaccination)	Unresectable Stage III NSCLC	Phase I/II study, single arm. Safe	[99]
EGF vaccine (day 1, 14 then monthly after completion of Cis/Vin chemotherapy)	Cyclophosphamide (200 mg/m^2^ 3 days before first vaccination and before monthly vaccination) Cisplatin (100 mg/m^2^) + vinblastine (6 mg/m^2^) once every 21 days for 4–6 cycles		Advanced NSCLC	Phase I study, single arm. Safe. Median survival better than previous reports.	[91]
Personalized peptide vaccine (once/ week)	estramustine phosphate (280 mg/day, p.o.; continuous)	Castration resistant prostate cancer	Phase II study, 2 arms comparing vaccine + low dose chemo to standard dose chemo. Median PFS in chemo/vaccine combo group was significantly longer than standard dose chemo alone	[100]
TG4010: rec. viral vaccine expressing MUC1 and IL-2 (once per week for 6 weeks, then once every 3 weeks)	Cisplatin (100 mg/m^2^ on day 1) + vinorelbine (25 mg/m^2^ on day 1 and 8; up to 6 cycles) - chemo given during or after vaccine therapy	Advanced NSCLC	Phase II study, 2 arms, historical control. Patients that developed CD8^+^ T cell response to MUC1 correlated with better survival;	[96]
DC-CAP-1 peptide vaccine (days 4, 10, 17 – first cycle only)	8 cycles of: Capecitabine (2000 mg/m^2^ PO per day days 1–14) + oxaliplatin (130 mg/m^2^ on day 1)	Stage III colon cancer	Phase I study, single arm. Evidence of increased T cell proliferation.	[101]

**Table 3.** Mechanisms of chemotherapies that could be used with cancer vaccines

**Mechanism**	**Chemotherapy**	**Ref.**
Increase Effector T cell Stimulation	Cyclophosphamide	[78]
	Paclitaxel	[49]
Increase Tumor Immunogenicity	Doxorubicin	[102]
	5-Fluorouracil	[103]
	Cisplatin	
Decrease Tumor Induced Immune Suppression	5-Fluorouracil	[64]
	Cyclophosphamide	[44]
	Gemcitabine	[62]
	Paxlitaxel/ carboplatin	[87]

**Table 4.** Immune modulatory monoclonal antibodies in development for humans (adapted from [110]).

**Target**	**Expression**	**Human Antibodies Available**	**Type**	**Development Stage**
**CTLA4**	Activated T cells	Ipilimumab (Bristol-Myers Squibb)	Fully human IgG1	Phase III complete
		Tremelimumab (Pfizer)	Fully human IgG2	Development halted after Phase III
**CD25**	Tregs, activated T cells	Daclizumab (Hoffmann-La Roche)	Humanized IgG1	Phase III
**PD-1**	Activated T cells	CT-011 (CureTech)	Humanized IgG1	Phase II
		MDX-1106 (Bristol-Myers Squibb)	Fully human IgG4	Phase II
**CD137**	Activated T cells, Tregs, NK cells, NKT cells, DCs, neutrophils and monocytes	BMS-663513 (Bristol-Myers Squibb)	Fully human IgG4	Phase II
**GITR**	Tregs	TRX518 (Tolerx Inc.)	Humanized IgG1	Phase I
**CD40**	DCs, B cells, monocytes, macrophages	Dacetuzumab (Seattle Genetics, Inc.)	Humanized IgG1	Phase I

## Abbreviations

MDSC: myeloid-derived suppressor cells
APC: antigen-presenting cell
Treg: regulatory T cell
DC: dendritic cells
MHC: major histocompatibility complex
CPA: cyclophosphamide
DX: doxorubicin
GEM: gemcitabine
PX: paclitaxel
mAb: monoclonal antibody
